# Border villages and the suffering of Kolberi: focus on Kolbers mothers’ narratives of their children’s Kolberi experience

**DOI:** 10.3389/fsoc.2024.1344806

**Published:** 2024-06-28

**Authors:** Hossein Daneshmehr, Kamal Khaleghpanah, Parviz Sobhani, Susan Rostami

**Affiliations:** Faculty of Humanities and Social Sciences, University of Kurdistan, Sanandaj, Iran

**Keywords:** border village, border residence, Kolberi, lived experience, interpretive phenomenology

## Abstract

**Introduction:**

The present research investigates the activity of Kolberi in the border villages of western and northwestern Iran, specifically in the Kurdish area of Nosud in Kermanshah province. Kolberi, a form of labor in these regions, subjects individuals to severe risks, including painful death or lifelong physical injuries, bringing considerable suffering and hardships to the Kolbers and their families. This study explores the narratives of Kolbers’ mothers regarding their children’s Kolberi experiences through Pierre Bourdieu’s theoretical framework of social suffering.

**Methods:**

This qualitative study employs interpretive phenomenology to examine the lived experiences of mothers in the Nosud border area. Twenty-two Kolbers’ mothers were selected using purposive sampling. Data collection was conducted through semi-structured interviews and participant observation, continuing until theoretical saturation was achieved.

**Results:**

Content analysis of the interviews revealed eight basic themes: (1) occurrence and aggravation of physical and mental complications, (2) reproduction of poverty and misery, (3) marginalization of the field of education in border areas, (4) emergence of structural determinism alongside environmental determinism, (5) weakening of the social status of Kolbers, (6) Kolber and bare life, (7) structural dehumanization of Kolber’s position, and (8) unique experiences of mothers regarding Kolberi.

**Discussion:**

The findings highlight the unique and often neglected experiences of mothers related to Kolberi, emphasizing the economic struggles in Iran’s border areas. These experiences unveil hidden aspects of Kolberi, suggesting potential avenues for further research and contributing to the revitalization of activism among Kolbers’ mothers in border regions. The study underscores the importance of addressing the socio-economic conditions that perpetuate Kolberi and its associated sufferings.

## 1 Introduction

Kolberi in Kurdish refers to border work in which young and old workers who are called Kolbers carry goods on their shoulders and cross the borders of Iran, Iraq, and Turkey. This very dangerous and risky work is often accompanied with many threats for the Kolbers, including the danger of death due to border guards shooting, with the threat of mines left over from the war-time of Iran and Iraq, falling from deep valleys and difficult mountainous places, and freezing in the snow and blizzard of winter (Soleimani and Mohammadpour, 2019). Kolberi now considered as a job in the western and northwestern border provinces of Iran (West Azerbaijan, Kurdistan, and Kermanshah), the job of young people and even the older ones who are in absolute unemployment on the outskirts of cities and villages. They have lower levels of economic capital and they inevitably turn to Kolberi, which is referred to as a kind of smuggling in the official discourse of Iran. In fact, Kolber is a creature similar to *Homer’s Sisyphus*, who moved a huge stone, rolled it, and carried it uphill more than a 100 times. Kolber-Sisyphus has a crumpled face, clinging tightly to the stone, with shoulders bent under the weight of the load and cracked hands. He carries with him a deep suffering caused by repeating the cycle of futility. This stone, or better to say, the bag falls from his hand many times and returns to its original place (sign of the peak of inferiority and misery) and he inevitably has to repeat this process again. In other words, Kolber is suspended in a dialectic of existence and non-existence, living and not living, a desperate attempt to survive in a life full of pain, suffering and blood (Mohamadi et al., 2021).

Border-dwelling is a form of social life that is based on fluctuation and contradiction and constantly instills a sense of temporariness, rootlessness, and marginalization to its residents. In fact, the border dweller is different from the centrist subject. The social life of this other, is always affected by the political economy ruling the border areas and with stereotypes such as the constant risk of insecurity, the lack of investment opportunities, the local culture as the main obstacle to development, the existence of ethnic prejudices, and the inherent tendency of people to make a living through smuggling of goods (Sohrabi, 2017). These stereotypes also form the main structure of the discourse and development policies in the border areas and play a key role in the construction and reproduction of the existing situation and also the main obstacle to a deep and methodical understanding of the methods and forms of life. These people live in border areas and are regarded as the “others.” It is not possible to recognize the border dweller as an “other” who has a different life from the center dweller, without neglecting from these stereotypes (Mohamadi et al., 2021). More particularly, this notion of being classified as “others” or experiencing otherness is more boldfaced as far as the Kurds are concerned (Alinia, 2004).

Border areas, being on the outskirts and far from the city center, tend to have limited resources and social services. This can result in poverty, lack of schools, economic struggles, and push people into smuggling and illegal trading across the border (Sadeghi et al., 2014). Border residents often make a living through illegal trading or smuggling activities. Living near international borders means they have a lot of business opportunities with neighboring countries (Hernández, 2023). But it’s the case to know about the definition of smuggling at first. Smuggling is an economic activity that is politically defined and socially embedded. In its functional essence, smuggling is typically trade, anchored in the demand for certain products and the costs of their movement. At the same time, it is segmented from legal trade through laws, which are, along with their enforcement, deeply political, tied into processes of state formation and demarcation, economic regulation and prohibition, and geopolitics and conflict. Unlike most trade, smuggling in its perception and study is also intimately tied to the figure of the ‘smuggler’ and the particular social space of the borderland in which they are imagined to operate – as a risk-taker, a broker, a hustler, a worker, a profiteer, a villain, or a local hero. We define smuggling as the purposeful movement across a border in contravention to the relevant legal frameworks. It should be clear from this that smuggling, as an activity and as a field of study, is fundamentally politically defined. Both the borders that make smuggling cross border trade and the laws that make it illegal are social and political constructs. This of course means that the boundaries of smuggling are movable and embedded as much in the context of an activity than in the activity itself. Critically, they are conditional not just in space but also in time (Gallien and Weigand, 2021).

According to Lee (2015), Unauthorized trade may be called smuggling by the government, but for Burmese merchants living along the riverbank, it’s just a normal part of life. Local authorities even acknowledge it. These merchants make a conscious choice to smuggle goods as a way to make a living, and the smuggled items are essential for the local community and play a big role in the economy of border towns.

The lack of development of the border areas has forced the border dwellers to turn to Kolberi, resulting in a large number of people, both men and women, old and young, to accept the risks associated with this work to earn their living. With the ever-increasing expansion of this type of business in recent years, various activities have been carried out by the government to deal with this phenomenon. These activities include human and electronic blocking of the borders through setting up border patrols, monitoring the border strip with the use of electronic equipment, and identifying and arresting Kolbers as well as confiscating their property and sometimes armed confrontation with them. In spite of the measures taken, we are witnessing the boom of the Kolberi market and the growing desire of the border residents of this region to this phenomenon (Salimi and Ghodrati, 2020).

Given that the attitude towards border areas is a top-down view and is often summed up in a security view, the development and progress of these areas in the official discourse is more like a propaganda and far from reality. This is where the official narratives of the border strip and geopolitical analyses are based on the supposed security vacuum in these areas, and this thinking has also led to the underdevelopment of these areas and the necessity of the border residents to turn to Kolberi. Unemployment as one of the most important issues in Iran is estimated to be 10.96% in 2022 (O’Neil, 2024), which is more severe in the border areas. In their research, Zare Shahabadid et al. (2020) came to the conclusion that 61% of the livelihood of the border dwellers is dependent on the border and border trade (especially smuggling and Kolberi). 86% of the border dwellers studied in the research believed that closing the border could have a negative impact on their economic situation. Therefore, the sociological view of this phenomenon and how it is reproduced among the border dwellers and its social consequences for families is of great importance. Kolberi is one of the high-risk occupations which, in addition to individual consequences such as various diseases, harms and physical-psychological pressures, also have social, economic, and political consequences. Among the problems and injuries faced by the Kolbers are various types of muscle pains caused by carrying heavy loads on their shoulders and crossing dangerous mountain paths, getting caught in unneutralized minefields, and death or amputation as a result of mine explosions in border areas (Sobhani and Daneshmehr, 2024). Daneshmehr and Mahmoudi (2023) in another work concluded that there are some other calamities that will affect the lives of the border dwellers; something like weakness of facilities, lack of suitable infrastructures, marginality of environment and the threats of border relations. The internalization of poverty and its economic consequences in Kolber-landing provinces and regions including smuggling, and finally the political consequences of smuggling, causes border crossing, resistance to official discourse, and disregard for political issues. And also Kolberi as an activity boosts the economic gap between borderlands and the other areas of the country. This also has led to the fact that the phenomenon of Kolberi is considered as a rooted occupation in border areas that has caused a lot of bodily, social and psychological harms of the occupants. This reality has brought the subalternity of this occupation in comparison with the others occupations of citizens in the other parts of Iran. Therefore, center-dwellers in Iran nominate Kolberi as a difficult and inhumane job that has caused discontent among border dwellers who believe they have been ignored economically. This point is of greater significance when growing number of children and teenagers are ready to carry heavy loads on the difficult roads and impassable mountains of the western border of the country. Although these data have not been officially published, the field investigation of the border villages of Kurdistan, West Azerbaijan, and Kermanshah provinces shows that the population of Kolbers living in these areas has increased significantly and is about 70,000–200,000 (Yoosefi Lebni et al., 2023).

Although the dangers and sufferings caused by Kolberi concern the Kolbers directly, their family members, especially their mothers also bear a lot of pain and suffering, which is not represented except in cases of their children’s death. Kolberi is always associated with stress and worry for Kolbers’ mothers as the closest and most grieving person to these people to the extent that it affects their daily life. These mothers sometimes even spend many nights waiting for their children until morning. Kolbers’ mothers always experience life with anxiety and fear from the time their children leave for the border and carry load: the fear of their children crossing the impassable mountains in the dark of the night, the fear of seeing the bodies of their children shot or dismembered due to the explosion of landmines left over from the war, the fear of their falling from cliffs and the resulting dangers, and the fear of getting lost in the winter snow as well as rain and freezing in the blizzard. But why mothers? And why is the suffering and hardship of other members of Kolbers’ families not addressed in this research? It is mostly because a mother gives birth to a child, with a thousand hopes and dreams and with the hope of her child’s growth and progress, she raises him and has many dreams for him that external determinism, the necessity of livelihood, the need for bread providing for the family and many other necessities causes mothers to give up their dreams for their children and forcefully agree to their employment in Kolberi. In many Kurdish folklore poems and songs, many have talked about the heartbreaking and sorrowful songs of mothers mourning their children who were killed while working as Kolbers or defending their land and beliefs. Therefore, it is because mothers are always worried about their children and are constantly waiting for impressive news from them. Of course, this does not mean that other family members such as father, brother, wife or sister is indifferent to this issue, but the grief of mothers is much different and deeper. Therefore, there are a lot of research on Kolberi from their own viewpoint narrating their sorrows and sufferings, but this work has been done far from this narrations and focus on the sorrows of the other members of the family, especially their mothers.

Among Kurdish women, the concept of return is a maternal thing; this issue is institutionalized in the social, economic and political context of Kurdistan society; because men are engaged in activities outside the home and due to the lack of employment opportunities, they generally live in non-fatherland. These are the mothers who are looking for a kind of expectation and peace and stability in the society and have always been the guardians of property, language and land; So that the protection of these cultural and social pillars has created a close link with political identity.

Iran shares a 1,458-kilometre-long border with Iraq, of which some 500 kilometers pass through the Kurdistan region.1 Smuggling of goods across this border has been happening for decades. Over time, this illicit trade has become consolidated, and operated by well-organized criminal networks consisting primarily of businessmen on either side of the border, who deploy smugglers or delivery drivers who transfer goods to collection points in the mountainous border regions, and couriers who carry the goods by foot or on horseback along the last stretch of the route across the border into Iran. Smugglers and couriers are exclusively Kurdish, either of Iranian or Iraqi origin. Many of the couriers have been plying their trade across this border for decades, and some have passed on their knowledge of the routes and systems to their children, and work as family units. The goods-smuggling trade from Iraqi Kurdistan to Iran is highly organized. It primarily consists of so-called grey-market goods that have been legally imported into Iraqi Kurdistan for local market consumption, but which are then redirected to Iran, largely evading official export or import procedures and duties. Alongside this grey market, concurrent networks facilitate the transportation of black-market goods – notably alcohol, which is illegal in Iran, and cigarettes originating in the West, the sale of which is prohibited in Iran in an attempt to protect the local industry. The most criminal aspect of smuggling in the region, however, involves the cross-border movement of weapons, narcotics and raw materials for manufacturing drugs, which are smuggled across the Iraq–Iran border using broadly similar methods and routes as deployed for the ‘grey’ goods supply chain. In the remote mountainous areas, the collection points are effectively makeshift base camps, where goods are delivered by Iraqi smugglers or their delivery drivers. Here, large quantities of continually replenished stocks of contraband are guarded by Iranian Kurds, as many as 15 in some places, who stand guard over the supplies, sometimes for months at a time. Their wages are paid by one main smuggler, funded by a financial deal brokered by smugglers on both sides of the border (Global Initiative Against Transnational Organized Crime, 2019). This is a part of neoliberalism program that is run in the area of Kurdistan through non-development and the non-interval of Iran to world order that is represented in this way.

Given this situation, no governmental or non-governmental organization supports them, and it seems certain that their only supporters are their families. In fact, the burden and responsibility of a Kolber, who has lost his life on this route or suffered amputation and other physical injuries, is on his family. The family of the killed or injured Kolber must also take care of his children. Because of the poverty and lack of necessary livelihood, the small children of the family inevitably turn to Kolberi and this is where the cycle of Kolberi continues and the sufferings caused by it continue to be produced and reproduced. The suffering and pain caused by this cycle mostly affects mothers. Against this backdrop, the current research seeks to explore the lived experience of mothers whose children experience such exhausting work continuously. The field studied here is Kolbers’ mothers in the border town of Nosud in Paveh city; located in Kermanshah province in the west of Iran. Since this city is one of the most deprived areas of the country in terms of development and also due to the fact that it is located five kilometers from the country of Iraq, it has become one of the most important cities for Kolberi. Nosud shares 42 km border with Iraq. Halabja is one of the most important cities of Iraq in the neighborhood of Nosud, which is located on the road between Sulaymaniyah and Kirkuk. The cities of Bayare and Tuileh are located in the west of Nosud and in the east of Halabja in Iraqi territory, and the border of Biareh and Tuileh is the most important border for Kolberi to Nosud. Due to the proximity and ease of access to Kurdistan Reign of Iraq in comparison to the city of Kermanshah, the residents of Nosud region prefer to provide their general needs from there and neighboring border villages. With this definition, the present research seeks to answer the following questions: (1) How do the studied mothers interpret the phenomenon of Kolberi and how do they feel about it? (2) What is the meaning and concept of children’s Kolberi, according to their mothers? (3) How do mothers see Kolberi as a problem through their children’s occupation? (4) What changes have the mother’s interactions undergone in the condition of the children’s health?

This study delves into the underexplored complexities of the border along Iran, shedding light on a unique and understudied site. Unlike conventional border studies that often concentrate on migrants or refugees making one-directional journeys, this research offers novel insights into the experiences of workers and couriers in the border region of Iran. The empirical focus is on Kolbers, Kurdish porters engaged in continuous cross-border activities, traversing the boundaries between Turkey, Iraq, and Iran in their daily working lives. This distinctive perspective captures the essence of ongoing exposure and challenges faced by these individuals, emphasizing the nuances of sustained engagement with the border. The border region in question emerges as a dynamic space where the continuous movement of workers and couriers exposes them to various forms of border violence and inherent dangers. This unique vantage point contributes a fresh dimension to border studies, departing from the conventional migrant/refugee-centric narratives. By centering on the experiences of those engaged in ongoing border-crossing activities, the research unravels the intricate processes and inequalities embedded in the daily lives of individuals navigating the complex terrain of the Iran border region. This emphasis on continuous exposure and the recurrent nature of border-related challenges enriches our understanding of the lived realities of those operating within the border rather than those moving across it.

## 2 Definitions and theoretical framework

Job acts as a kind of mutual identification model, with the help of which one can reach the needs and abilities as well as the social and economic status of individuals. Job actually includes certain key information such as income, status, linguistic abilities, potential interests, social connections, and so on (Beck, 1992). In the developing countries, in which a vast majority of the world’s young people are living, numerous youths are poor people who are more likely to have informal jobs under unfavorable working conditions. Nevertheless, as we have seen recently, youth unemployment is not only a challenge of underdeveloped countries but also it is a global challenge which also affects developed countries. The unemployment crisis might have enduring, detrimental outcomes for the unemployed young people. Although unemployment is not favorable at any age, it is more adverse when it happens early in an individual’s work life (Vogel, 2015). Unemployment in border areas is compensated through getting down to smuggling as a job. In some border areas in which there is no hope to change and development, this kind of job will become stabilized in the minds and activities of the border dwellers.

### 2.1 Informal economy and inappropriate employment

The informal economy, consisting of practices which possess market value and would contribute to tax revenue and GDP in case they were recorded, is regarded as a widespread event over the globe. In accordance with the International Labor Organization, about 2 billion workers or 60 percent of the employed people whose ages vary from 15 to older ages spend at least part of their time in the informal sector. When economies are developing, the size of the informal sector might gradually decline. However, this change varies in various regions and countries across the globe. Nowadays, the informal sector still comprises approximately a third economic activities of low- and middle-income countries—the portion which equals 15 percent in the advanced economies. From this perspective, Informality includes a broad spectrum of contexts within and across countries and it is still expanding for various reasons (International Monetary Fund, 2020).

The informal economy consists of a wide number of basically various activities that are carried out for various causes. The production, distribution, and consumption of illegal goods like narcotics fall on one extreme. But not all informal practices are illicit. Ordinary goods or activities that are kept from the government make up a significant portion of the informal economy. Hiding can be used either as an active strategy to counteract corruption and rent seeking or as a passive rent-avoidance strategy to escape an influence group-captured government. At the other hand, due to either government failures in economy registration and control or the underdeveloped and small scale of these irregular activities, there are activities that are not hidden but remain unobserved (Khandan, 2017).

Many researchers have directed significant research attention to the abundance of the informal works and its widespread emergence. The existing literature could be predominantly categorized into two theories. The former theory, based on the International Labour Office (1972), views the informal economy as a distinct market where poor individuals are working only for survival purposes. This theory, known as “dual labor market” (DLM) or “labor market segmentation,” investigates the impact of immigration, citizenship, gender, education, and other causes of discrimination on workers’ engagement in formal and informal sectors. Based on this theory, the informal economy expands if the formal economy does not to provide sufficient jobs to attract all labor force. However, of the majority of the studies conducted so far have been predominantly descriptive, concentrating on the features and distinctions of various sections and mobility existing across both formal and informal sectors (Pagés and Stampini, 2009; Hudson et al., 2012; Lehmann, 2015). The DLM theory is concerned with the quality of informal works as well as the health and wellbeing of individuals who are forced to do these jobs. According to this theory, policies which can increase productivity in the informal economy should be employed (Kantor et al., 2006; Lehmann and Pignatti, 2008; Haanwinckel and Soares, 2021). Understanding the competition between formal and informal economies to attract workers and the impact of the macroeconomic condition on the substitution of production factors between the two sectors is made easier with DLM. But it fails to elucidate the illegal motivations involved in numerous illegal and hidden activities. The informal economy plays a significant role in meeting the financial needs of families with low incomes in developing nations like Iran. There are over six million informal jobs in this country, and Kolberi is one of the most prevalent in many border Kurdish-populated Iranian cities (Mottaghi et al., 2022).

### 2.2 Border and bordering

Historically, borders, as lines outlining two countries, have been examined and considered as geographical indicators of regional control, state power, and signs of public characters. Borders have become an important part of how states approach security, power, and wealth over the centuries (Bissonnette and Vallet, 2020). Nowadays, borders are widely acknowledged as intricate, multilayered social phenomena connected to human psychology and the fundamental organization of society. However, this has not always been the case. In other words, how borders have been examined and understood has developed much in consistent with broader discursive changes in social studies and with regard to underlying geopolitical phenomena (Paasi, 1999).

Urban informalities is a global real phenomenon which is evident in the cities of the developed as well as developing countries. Street trading and slum housing are two examples of informal spatial, political, and economic practices that fall under the umbrella of urban informality (Banks et al., 2020). Moreover, urban informality can be described using a variety of different concepts. Which consist of the informal sector, informal economy, and informal employment although such concepts indicate various meanings. For example, the informal sector is conceptualized as production as well as employment which occurs in unregulated or unregistered enterprises, whereas informal employment is concerned with any kind of employment that is without social and legal protection. Ultimately, informal economy deals with various informal economic sections, informal economic practices, and informal workers (Chen, 2012). Nevertheless, the focus is on Urban Economic Informality (UEI) in this article and the concept of UEI is employed interchangeably for the urban informal economy. Urban economic informality, in most cases, is conceptualized as informal economic activities which are carried out for the objectives of earning a living for survival as well as profit, or a combination of the former and the latter; nevertheless, these economic practices might not correspond with laws pertaining to production as well as distribution rules (Moyo and Gumbo, 2021; Thulare et al., 2021). According to other definitions, urban economic informality is characterized by less productivity, informal enterprises, informal economic practices, and unregulated labor (Damayanti et al., 2018). Nevertheless, more recent conceptualizations of urban economic informality include a larger statistical component that takes into account the size of the business and, in some cases, labor rights (Chen, 2012; Chen and Carré, 2020; Rosaldo, 2021). Within the past decades, Investigations on the UEI demonstrate that informal economic practices throughout the world have played an essentially significant role in offering socioeconomic livelihoods including job provision, poverty eradication, and decreasing the degrees of inequalities (Radchenko, 2017; Banerjee and Goswami, 2020). For example, in European countries such as the United Kingdom and Italy, the UEI has been a part of the economic building blocks towards employment creation (Koff, 2015). Likewise, in Sub-Saharan Africa, urban economic informality has been considered instrumental in affecting numerous poor societies concerning job creation, poverty eradication, and the overall improvement of living standards (Lanchun and Mingyu, 2019). Numerous studies have shown that the UEI has played a crucial role in affecting the characteristics of many emerging economies in the Global South countries (Sassen et al., 2018; Polese, 2021).

Smuggling economies undertaken by the Kurds are usually deemed as a legitimate way of earning among communities residing in border areas. The kurds are the predominant inhabitants in the both sides of the Turkish-Iranian border. This situation has made Kurdish communities of both sides to have familial, social, and economic bonds with each other across the so-called national border which has separated these people. Through imposing this official border, nevertheless, both Turkish and Iranian state officials have tried to regulate and confine the social and economic exchanges and relations existing between these border dwellers. Although state authorities have considered a number of these cross-border social and economic relations as illegal activities, Kurdish people have not given up to sustain these exchanges. From this perspective, the majority of Kurdish communities in the borderlands have viewed the cross-border trade activities that state authorities have criminalized and referred to as smuggling as socially and economically legitimate (Bargu, 2019).

Taken together, understanding the concept of borders within the context of this research is pivotal for unraveling the complex dynamics of border villages and the experiences of Kolbers. Traditionally viewed as demarcations between countries, borders have evolved into multifaceted social phenomena intertwined with historical, psychological, and geopolitical dimensions (Bissonnette and Vallet, 2020). The contemporary understanding of borders aligns with broader discursive shifts in social studies, reflecting a departure from mere geographical indicators to intricate manifestations deeply embedded in societal structures (Paasi, 1999).

One crucial facet of border regions, particularly relevant to this study, is the phenomenon of urban economic informality (UEI). UEI encompasses informal spatial, political, and economic practices evident globally, including street trading and slum housing (Banks et al., 2020). It entails various concepts such as the informal sector, informal economy, and informal employment, each carrying distinct meanings. For instance, the informal sector pertains to unregulated or unregistered enterprises, while informal employment lacks social and legal protection. Urban economic informality, in the scope of this article, refers to informal economic activities undertaken for survival or profit, often not aligning with established production and distribution rules (Chen, 2012). The complexities of border regions extend to smuggling economies, particularly observed among Kurdish communities residing along the Turkish-Iranian border. Despite official efforts to regulate these cross-border interactions, Kurdish communities perceive such activities as legitimate, fostering familial, social, and economic bonds transcending the imposed national border. This nuanced exploration of borders and border-dwelling lays the foundation for a comprehensive understanding of the lived experiences of Kolbers and their families in the border villages, aligning with the thematic focus outlined in the special issue’s call.

### 2.3 Kurdistan, border and smuggling

Whether it’s about land or politics, borders are a big deal because of the people they separate. Basically, they are like the main tool for sorting things out, like socially, politically, or ideologically. Setting boundaries has been a thing since forever, with individuals, groups, societies, or political bodies drawing lines to define themselves. But nowadays, drawing borders based on land is a pretty modern concept, and those strict, clear lines are mostly a thing for modern nation-states (Tekin, 2016). The scholarship on Kurdish history often talks about where Kurdistan is on a map or as a political idea, but it does not say much about how Kurdistan came to be and how it changed over the years. Over time, Kurdistan’s borders and its heart have shifted a lot, even its name has changed. It is a kind of “political geography” that’s shaped by society and history, looking at how people saw Kurdistan change, including its borders, landscapes, and cities (Alemdaroğlu and Göçek, 2023).

One of the important consequences of the collapse of the Ottoman Empire is the division of Kurdistan. The clash between the imperialist forces of the period resulted in a “consensus” about the division of Kurdistan into three parts. The emergence of three different economic units (Turkey, Iraq, and Syria) from a single economic unit destroyed economic relations based on empire logic. In short, the economic integration of Kurdistan during the Ottoman period and the administrative reforms initiated in this period, have gone to a new stage after the division of Kurdish lands by “national borders.” Each economic unit, or more precisely each new nation-state entered into an effort to create “a national economy” within its own borders. But the case was a little different about the Kurdistan of Iran that from the very beginning integrated in the Iran land. In this case it was done a lot to integrate this part of large Kurdistan in the lands of Iran by Iranian states and governments (Yıldırım, 2017).

People smuggling is a way for marginalized groups, like the Kurds, to overcome their struggles with ethnic conflict and poverty. Despite the dangers involved in these secret journeys, bribes and corruption help smugglers lower the risks and improve their chances of getting across the border successfully. While governments condemn this behavior, corruption is seen as a way to level the playing field for marginalized Kurds and help them survive in challenging border areas (Augustova and Suber, 2023).

Even though defining borders or border relations are not contemporary matters of fact, they have gained new senses and have been rebuilt with the former state forms leaving their place to other forms—especially with the appearance of a new understanding of hegemony. In today’s world, social, economic, and political life cannot be regulated without boundaries. When we think about border and economic relations, illegal trade (“smuggling”) expressing a disruption especially for the states confronts us. Borders and smuggling have been defined relative to each other to a certain degree. By definition, for smuggling to be identified as such, there has to be borders and the state has to declare which goods are allowed to be imported and exported legally. Therefore, the illegal evaluation and punishment of the commercial rituals between any two regions, called “smuggling,” came into existence with the emergence of state borders. In this context that smuggling is a crime against the state rather than a crime committed against the individual or against his/her property. According to him, smuggling is not observed in small-scale societies, which can take measures for the flow of illegal goods and lack the common and legal expression of social interests. On the contrary, smuggling is only found in political units organized in the form of state. Cross-border trade, which is accepted as illegal by the legislation, may only be an extension of existing trade. However, smuggling is often the result of restrictive state policies, which cause some goods to be scarce, attractive, or expensive (Yıldırım, 2017).

### 2.4 Bourdieu and social suffering

The concept of social suffering was first used by Pierre Bourdieu in the book “The Burden of Social Suffering in Contemporary Societies.” The pain and suffering that Bourdieu’s sociology portrays here is not limited to the misery of living conditions caused by the lack of facilities and material as well as financial poverty of people, but it shows new forms of misery in which the legitimate goals and aspirations of people in the way of happiness and personal growth is constantly bumping into strong obstacles and coming up against rules for which individuals are not prepared. Bourdieu reveals the barriers that are produced and reproduced in the home, school, and labor market, as well as tough social competition and urban violence. He also depicts the mechanisms that make life painful and even unbearable (Bourdieu et al., 1999).

Emphasizing the historical nature of the economic field, Bourdieu shows that the social issues cannot be reduced to the economic issues. The social issues, which include culture, education, economy, politics, symbolic matter, and so on, simultaneously affect each other and cannot be analyzed independently (Bourdieu, 2005). The field is a base of conflicts, a kind of socially constructed base of action where actors with different resources face each other to maintain or transform existing power relations (Bourdieu, 2004).

The field is a structured social space or a field of forces in which we deal with powerful people with continuous and stable relationships as well as inequalities that occur within this field. This field is also a base of struggle to change or maintain the field of struggle. Everyone in this world is engaged in a competition, the subject of which is to obtain more relative power in his hand, leading to identifying his position in the field and his strategies accordingly (Bourdieu, 1996).

In order to fully understand the place of suffering in addition to economic policies, Bourdieu tries to consider the concept of symbolic violence as the other side of the coin of suffering production. According to Bourdieu, the roots of pain and suffering cannot be recognized by the members of society in symbolic violence, and people themselves internalize the foundation of the production of suffering. This is done by those who apply it since both groups are aware of its actions (Bourdieu, 1996). From Bourdieu’s perspective, the hierarchies or various forms of social inequalities in today’s society, which cause human suffering and hardship originate from the forms of domination rather than from the relations related to the exercise of power. The result of this type of domination is symbolic violence. The theme of symbolic violence is the result of Bourdieu’s understanding of language. He considers language as an expression of power and action. He believes that hierarchy gives order to the world and places people in categories and arranges them. Political conflicts are an attempt to legitimize the classification system. From this point of view, symbolic violence is the unknown and imperceptible state of violence in its general sense because the dominant class needs the least energy for its domination. As a result, the dominant class is only engaged in doing the daily work. As long as the dominant and subordinate classes recognize symbolic violence, it will be reproduced.

Bourdieu is one of the contemporary theorists who places special emphasis on lived experiences among activists and considers the importance of narratives in various fields. In his recent book titled “Weight of the World,” he has full codes about experiences and interpretation. Therefore, in this research, the lived experience of a specific group (Kolbers mothers) in a specific field (border regions of Kurdistan) has been studied. Bourdieu despite his historical and theoretical disparities converges in his shared recognition of the profound impact exerted by social structures on individual agency. Bourdieu’s literary work, “The Weight of the World” (1993/2000), unfolds a tapestry of narratives featuring individuals grappling for survival amidst the constraints imposed by societal structures. These constraints, painted as natural, pose a threat to the social and cultural autonomy of the characters, embodying narratives of resistance and endurance against externally imposed conditions. These externally imposed conditions parallel the precarious existence of marginalized groups, exemplified by the Kolbers – Kurdish porters navigating the border between Turkey, Iraq, and Iran while carrying heavy loads. Kolbers find themselves in a state of limbo, torn between leaving their homeland in pursuit of economic opportunities in urban centers or remaining in their native regions despite the looming threat of peril. As a matter of fact, Bourdieu underscores the internal significance of the struggles, emphasizing the intricate interplay between societal structures and individual agency in shaping lived experiences.

## 3 Methodology

The method of the current research is phenomenological and interpretative research. Phenomenology has its pros and cons. On one hand, it gives a deep look into human experiences and the findings come straight from the data. But on the other hand, it relies on how well the subjects can express themselves and needs the researcher to interpret the results. The aim of phenomenological analysis is to break down the meaning and idea of phenomena. The aim of phenomenological research is to clearly explain and recognize phenomena, as they are seen by people in a particular situation. Phenomenology helps to get a better grasp of the nature and significance of everyday experiences. The main idea behind the phenomenological approach is to look for common threads in people’s experiences to understand and describe a particular phenomenon. Researchers talk to a bunch of people in detail to figure out what these common threads are. The purpose of a phenomenological study is to study the lived and shared experiences of a common group to identify a phenomenon. Bourdieu’s key concepts, including habitus, body hexis and doxa are closely related to the lived experience of people in a target society; Thus, their experiences of a specific phenomenon can be the result of the construction of this phenomenon in their social life that can be reproduced constantly. The reason for choosing this method for the current research is to explore the lived experience of Kolbers’ mothers and to understand the construction of the meaning of children’s Kolberi from their perspectives. Interpretive phenomenology has its roots in the works of scholars such as Heidegger, Merleau-Ponty and Sartre. They maintained that phenomenology is not just a description but an interpretation process in which the researcher interprets the meaning of people’s lived experiences (Van Manen, 1990). In the interpretive approach, there is a hermeneutic cycle, which means that there is a constant back-and-forth process between the researcher and the participant (Wajnar and Swanson, 2007, p. 175) (Table 1).

**Table 1:** Comparison of population statistics of villages in Nosud district, Kermanshah Province, Iran in 1975 and 2015.

	Village name	Population based on 1975 census	Population based on 2015 census	Population growth comparison
1	Neysaneh	204	229	3.12%
2	Tashar	309	306	−1.0%
3	Narvi	438	424	−3.2%
4	Hani Garmalah	1.402	869	−38.0%
5	Sharrakan	508	236	−53.5%
6	Wasli	276	115	−58.3%
7	Hajij – e Bozorg	1.041	321	−69.2%
8	Bidarwas	401	123	−69.3%
9	Zbar	37	11	−70.3%
10	Dzawar	1.257	289	−77.0%
11	Kaymnah	1.016	127	−87.5%
12	Shushmi Aolia	421	46	−89.1%
	Total	8.162	3.096	−62.1%

Source: https://www.amar.org.ir.

**MAP 1:**
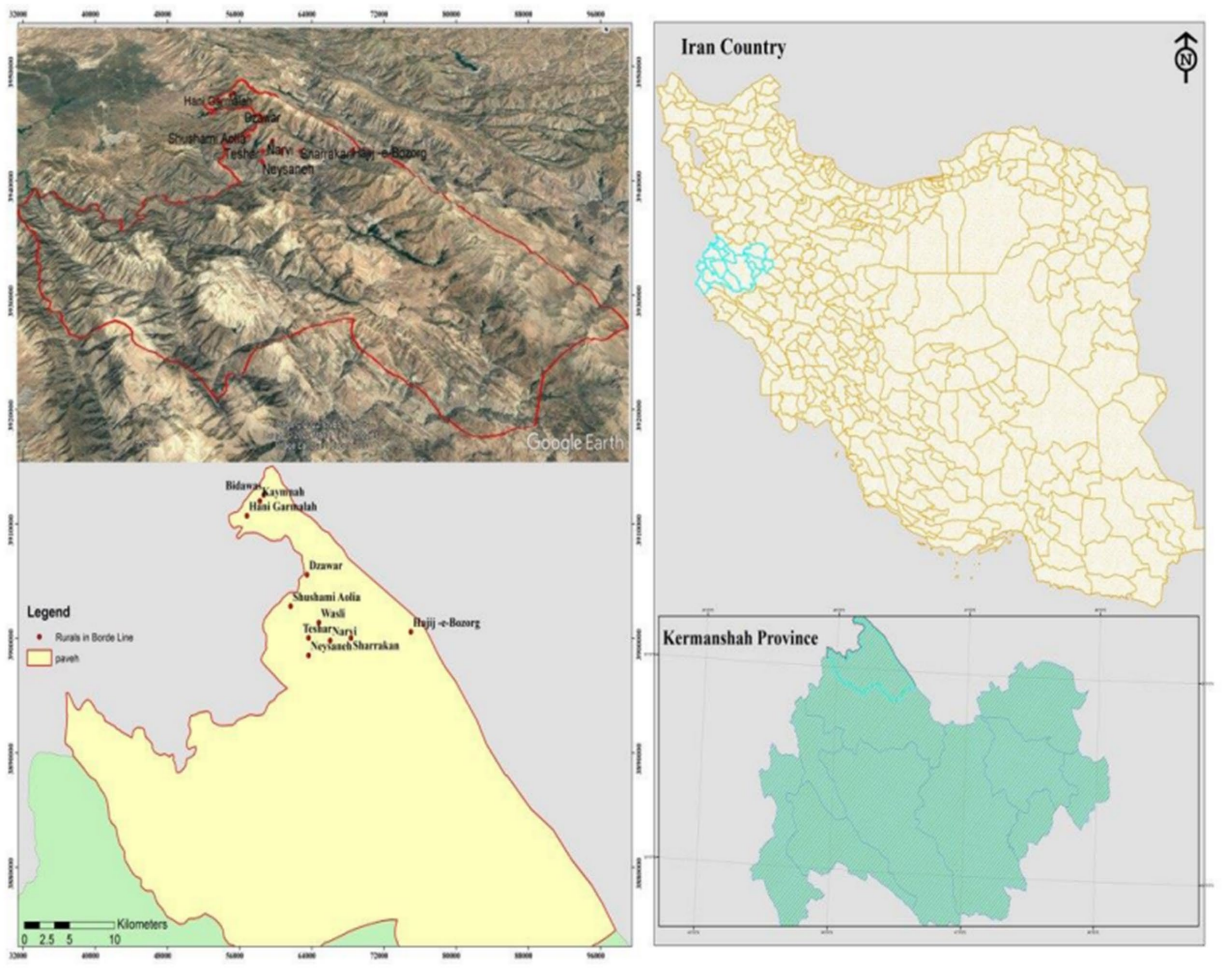
Geographical location of the studied area.

The sample includes mothers living in Nosud district; a district near the City of Paveh in the Province of Kermanshah, west of Iran. Nosud shares 42 km border with Iraq. One of the most important cities in Iraq which is in the neighborhood of Nosud is Halabja in the Kurdistan Region of Iraq, which has the most border relations with the residents of other border villages in this region. In this part of the article, the spatial appearance of the studied area is presented (Map 1).

The sampling method of the current research is purposive sampling and includes the mothers whose children are engaged in Kolberi and who live in the villages of Nosud sector. The people in the villages of Nosud region share a lot in terms of ethnicity, language, religion, and kinship. They also hold common rituals, which makes them more aware of each other’s lives, especially work and employment. Since researchers also live in this area, they have a deeper understanding of each other and their living conditions. As a result, people have a lot of information about each other’s lives in this region. Therefore, according to these conditions, it is easy to choose mothers whose children are engaged in Kolberi. Because mothers always talk about the condition of their children in the villages and in common ritual ceremonies among all the villages in this region, and in this way they sympathize with each other. In phenomenological research, the purposeful or criteria-based sampling strategy is used to select the desired samples and units (i.e., lived experiences). Based on this strategy, participants are selected according to their specific knowledge about the phenomenon under investigation. As such, the sampling continued until the data saturation was achieved. In so doing, 22 participants were interviewed.

Data collection was done through in-depth interviewing (see the Appendix) with the interviewees (Kolbers’ mothers). Living in the target area and society for a long time, the researchers could observe the condition there and have lived experience of understanding the situation. As cited by Cope (2003) stated that the purpose of phenomenological interview is to obtain a first-hand description of some special areas of experience, and they consider the role of the interviewer to provide a situation in which the participants feel the need to describe their experiences in detail. The credibility of the current research was obtained through interpretive validity. In this research, in order to increase the validity and reliability of the collected data, it was tried to consider issues such as the diversity of the participants in terms of the age of their children, the marital status of their children, the duration of their children’s marriage, the number of children. Regarding the data coding and trustworthiness, two experienced, independent researchers in the field of qualitative studies were consulted and asked to blind code some textual data. The demographic characteristics of the participants including gender, educational background, and their occupations are presented in Table 2.

**Table 2:** Characteristics of the interviewees.

Interviewees	Child’s age	Child’s educational background	Child’s marital status
Interviewee 1	35	Diploma	Married
Interviewee 2	25	Junior high school	Single
Interviewee 3	18	Senior high school	Single
Interviewee 4	24	Senior high school	Single
Interviewee 5	57	Senior high school	Married
Interviewee 6	30	Junior high school	Single
Interviewee 7	42	Junior high school	Married
Interviewee 8	27	B.A	Single
Interviewee 9	29	Diploma	Single
Interviewee 10	24	Senior high school	Single
Interviewee 11	28	Junior high school	Single
Interviewee 12	24	Junior high school	Married
Interviewee13	37	Junior high school	Married
Interviewee 14	40	Junior high school	Married
Interviewee 15	16	Senior high school	Single
Interviewee 16	18	Senior high school	Single
Interviewee 17	35	Pre-university	Married
Interviewee 18	17	Junior high school	Single
Interviewee 19	40	Junior high school	Married
Interviewee 20	26	Senior high school	Single
Interviewee 21	24	Junior high school	Single
Interviewee 22	32	Senior high school	Married

It is noted that each interview lasted from 1 to 2 h and the interviews were conducted in Kurdish to allow the participants to express their ideas eloquently.

It is also worth noting that the human images featured in this article were utilized with explicit permission granted by the participants themselves, with additional images adapted from local newspapers a process further validated and confirmed by the Research Ethics Committee of the University of Kurdistan.

## 4 Results and discussion

The thematic analysis of the qualitative data via data coding yielded eight intriguing themes as follows. The initial step in analyzing the themes involves a formal analysis of the text. Non-narrative phrases are removed, and the refined text is subsequently subjected to formal analysis. The following step entails the structural description of the content, utilizing thematic analysis as the technique. Thematic analysis involves identifying and examining basic ideas by coding the interview content (Strauss and Corbin, 1990). Thematic analysis and structural analysis are the two most commonly used methods in narrative analysis.

### 4.1 Occurrence and aggravation of physical and mental complications

Due to the logic of centralism in economy and politics and moving from the center to the periphery, poverty, unemployment, inequality and the difference resulting from the dialectic of centrality would increase. In the absence of the government’s attention to infrastructures and redistribution of wealth and resources, the residents of the border areas and especially those of Nosud have tried to use the border location for livelihood and to take new ways against the official-political structural pressures. One of these ways to make a living in the western border areas of the country is Kolberi. Because this work is often done by the person without using any other means, it is associated with many complications and physical problems for the person.

In this regard, a Kolber’s mother said: *“Kolbari is very dangerous and worrying. My son had an operation on his back disc because he was going to Kolberi and four vertebrae in his neck were broken.”*

Also, another Kolber’s mother said: *“Kolberi is a very stressful job. You are even afraid of an accident in the car with which you go to Kolberi, fear of running over a mine. War-torn areas where several people have been killed and disabled in them. However, no one has been responsible and nothing can be done.”*

Another interviewee said that: *“My son went for Kolberi and was shot, his hand was paralyzed…. one of my other sons went bankrupt with Kolberi. We sold everything to pay the debts. This is the future of Kolberi.”*

Passing through the narrow mountain roads, which is mainly done at night and away from any lighting to avoid attracting the attention of the border guards, causes many physical issues and problems such as back disc, bone damage of hands as well as feet, and the possibility of getting caught in minefields or slipping from the rocks and falling into the valleys. On the other hand, their mothers are constantly struggling with stress and anxiety caused by any issues that may happen to their sons from the time their children leave home to the time they come back. All of these have gone hand in hand and aggravated the physical and mental problems for the children and especially their mothers. There are a lot of dangers for the Kolbers, but the biggest one is when border guards shoot at them or when they get arrested and their stuff taken away. Despite all this, some Kolbers take the risk to move goods from Iraqi Kurdistan to Iranian Kurdistan for a fee. If anything goes wrong with the goods or Kolber, the cargo owner cannot ask for any compensation or damages. But often, Kolbers are paid double or triple to ensure that the goods they deliver will make it across the border without any issues. If something goes wrong (like the cargo being found and confiscated), they have to cover the full cost for the owner. This puts Kolbers in a tough spot, causing problems and hardships for them and their families.

Social capital is a key idea from Bourdieu that helps people navigate social interactions. According to Bourdieu, those who are marginalized are basically shut out from having any kind of influence, so they try to gain capital to be part of different social circles. The narratives of Kolbers’ mothers show that Kolbari is seen as a risky environment that has become a norm for those living in border regions with no other options. As a result, being excluded from economic, social, political, and cultural spheres has led Kolbars to rely on Kolbari.

### 4.2 Reproduction of poverty and misery

The majority of Kolbers are coming from poor, crowded, and low-income families in the border villages, whose geographical conditions such as the mountainous nature and the lack of supporting infrastructure in the fields of agriculture and animal husbandry lead them to turn to Kolberi in the border strip. Without having any capital, they only carry the loads of the owners in exchange for receiving a small amount of money. The main owners of the commodity are those who own the main capital and live in the City of Tehran. The second groups are the owners of capital in border areas that use Kolbers for carrying the goods. The most amount of profit belongs to the first group and the least for Kolbers.

According to the majority of them, poverty and lack of money have led them to this path.

A Kolber’s mother said: *“If we do not go for Kolberi, we will have to die of hunger, Kolberi is our only source of income. There is no job here, neither is agriculture and animal husbandry, which requires fertile land that we do not have. No one or no organization supports us.”*

Another Kolber’s mother said: *“With these difficult and expensive economic conditions, my son works as a laborer during the day and goes to Kolberi at night. My husband also has a family like this. If he does not go to Kolberi, our life will not spin.”*

Rural families in the border areas are forced to resort to Kolberi and bear the hardships due to the lack of social security and the weak support of some support organizations that do not provide for the livelihood of the families. Intensification of inflation in the whole country and the inability of related organizations in the field of supporting poor and destitute families has left no other way for them in the border areas. We even witness that this heightened unemployment and high prices in the past years have caused many young people from other central parts of Kurdistan and Kermanshah provinces of Iran to go to the border areas of these two provinces to work and earn money.

When Bourdieu was in Algeria, he looked into the hardships faced by the locals there. These hardships were mainly due to the authorities disempowering the native people. Bourdieu saw this suffering as a form of symbolic violence, where the larger systems worsened underdevelopment in those regions, leading to poverty, exclusion, and ultimately, more physical violence. This same pattern also affected those living in border areas, who also felt the impact of symbolic violence.

### 4.3 Intensification of maternal sorrows and hardships of Kolbers

Growing poverty along with factors such as hopelessness about the future of their children and the imminent harm to them has always added to the suffering of Kolbers’ mothers. This group of mothers, due to witnessing the situation closely and receiving daily news about the fate of their children, have always internalized sadness within themselves and struggle with it every day.

This time, a Kolber’s mother said: *“When my son goes to Kolberi, I only pray for him and I ask God to protect and return him safely. Not only for my son, but for everyone else. When I hear about something that happened to each of them, I say to myself, who is it and what happened to them? A mother worries about everyone, but for her children, more.”*

Another Kolber’s mother also said: *“When my son goes to Kolberi, I think about him a lot and I am constantly thinking about him, but I have to do the housework and wait for his return, and I count the minutes until that he comes back. I will be very happy when he comes back and I thank God that he is back.”*

Much news about the death of the Kolbers, their getting caught in the snow and blizzard of winter, or their falling into the valleys, or even their arrest by the border guards, all have a psychological burden on their mothers. Most of the mothers are aware of the fact that the harms and threats of Kolberi may befall their children at any moment, but they are forced to accept it because if there is no Kolberi, there will be no other way to feed the children’s stomach and meet the minimum daily needs. In other words, Kolberi is like a double-edged sword, both doing it and not doing it, is associated with issues and problems. Bourdieu was the first to talk about how people in traditional societies just naturally act and think, using the idea of common beliefs and phenomenology. In modern societies, common beliefs are those basic, clear ideas that come from our social circles. Kolbari is based on the border folks in western Iran, and those people are known for it.

### 4.4 The marginalization of the field of education in border areas

In spite of the need of children in remote and border areas of the country for education and especially quality education, there exist serious doubts about the ability of educational systems to create fundamental changes to reduce regional inequalities. The bulk of evidence shows that the general condition of schools in rural and border areas as well as remote areas is not very favorable for proper learning. Very weak facilities, untrained or inexperienced human resources, the internalization of indifference of teachers and students to educational topics, the lack of necessary intra-departmental and inter-departmental coordination, and the use of old educational methods are among the basic problems in this regard. For example, another Kolber’s mother said: *“My son left school and went to Kolberi. He fell behind in studies, he fell behind in culture, and he fell behind in everything. The reason is poverty and the lack of a suitable educational environment and motivation among our children because many of the graduates are also engaged in farming because of unemployment.”*

Another Kolber’s mother also said: *“My son studied until the twelfth grade, but due to the death of his father and poverty, he did not continue because he lacked money and he dropped out of school out of desperation.”*

Education in rural areas, especially border villages, has its own difficulties. In these areas, due to the lack of necessary educational facilities, the weakness of material and financial support for children’s education, the unemployment of educated people in the society and the severe financial constraints of border families, the desire to study and receive education is often associated with a high decline. Most of the students in these areas avoid education and prefer to follow the example of other family and village members and turn to Kolberi so that they can have financial savings for their future. This is where we face high dropout rates among mostly male students in the border areas because they mostly have a desire to go to Kolberi. The widespread and popular belief in Kolbari as the only field of representation in social relations has been institutionalized among the people of the western border regions of Iran. In fact, from Bourdieu’s point of view, Kolberi in border areas is the only field left for the agency and action of the border dwellers.

### 4.5 The emergence of structural determinism alongside environmental determinism in border areas

Most of the border areas in the west of Iran have a rough geographical texture, high and impassable mountains and rocks next to deep valleys and mostly cold weather and blizzards as well as snow in the cold seasons of the year. In addition to these issues, issues such as the lack of development of these border areas and their abandonment by the center and the lack of structural will and desire for economic, social and human prosperity in these areas have provided the ground for intensifying the peripheral deterrence. In fact, the lowest attention is given to the border areas in the developmental programs and the result is poverty and misery and the ever-increasing gap in their quality of life compared to the residents of the center.

In this regard, a Kolber’s mother said: *“Kolberi is only sadness and unhappiness and it is not like any other work at all. I lost two of my brothers in this way, my son was also paralyzed. Three years ago, my son damaged his back disc at the age of 17 due to Kolberi, although he underwent surgery, but he achieved his health again. It is very difficult for a mother to witness her child’s disability or the death of her loved ones.”*

Another Kolber’s mother said: *“Kolberi is a job that you really have to sacrifice your life for. Now that my son is going to Kolberi, there is no expectation that he will return, and we are waiting every moment for something to happen to him.”*

The current situation in the border area of Nosud, like other border areas in the west and northwest of Iran, is the result of a lack of planning, unwillingness, and lack of national will to solve the livelihood issues of the border residents of this region on the part of the statesmen and responsible structures. In fact, neglecting the development of the border areas and the lack of any effort to create productive employment pushed the border residents of these areas more and more towards Kolberi. As a result, Kolberi is a clear and direct consequence of the lack of development in the mentioned border areas. Structures are all about changing things up and controlling how people interact with each other. Bourdieu was all about this idea, seeing it as a way to control and dominate others using symbolic power. Symbolic violence reproduces itself in the border areas in the form of exclusion from the field of development and progress and enjoying the resources of wealth distribution. Kolbers are always facing this kind of violence in these areas on a daily basis and have accepted it.

### 4.6 Weakening the social status of Kolbers

Due to the fact that there is no hope for their work future and there are always various dangers and threats waiting for them, they lack any social status and capital. The official structure always looks at them as smugglers and subsequently deals with them accordingly. Even among themselves, they have reached this inner conviction that they have no individual and social status and are often under local and structural exploitation.

A Kolber’s mother said: *“Kolberi is not useful at all, it is done out of necessity and compulsion. There is no good view of Kolberi in the society and they look at them with a bad eye and call them smuggler.”*

Another Kolber’s mother said: *“Kolber is forced to go for Kolberi, because of lack of money, and misfortune. Kolber is oppressed with an uncertain fate.”*

A job can only bring social capital and legitimacy for its holder, when is along with high social acceptability and is a reliable point of support for its holder. Kolberi, with all its risks, is not recognized as a suitable job by the official structure or even by the social elites of the society. Even the Kolbers themselves never envisioned a social position or a future for it, but simply chose it as the last option and opportunity to earn a living, through which they could earn money, although it was accompanied by hardship and weak hope toward future. According to Bourdieu, the social status always depends on having social capital. In fact, due to not benefiting from capital (economic, social, political and cultural), the Kolbers have always been marginalized. They have tried to compensate for this vacuum by resorting to Kolberi and face all kinds of difficulties caused by it. Mothers’ narratives of these hardships are proof of the truth of this issue.

### 4.7 Kolber and bare life

Giorgio Agamben has a concept called “bare life.” He explains that Homo Sacer is a concept belonging to ancient Rome and was used to refer to someone who has committed an indecent act and violated one of the society’s taboos, and for this reason has been deprived of all his civil and human rights. In this way, Homo Sacer is a legal term that is also used in ethics. In this way, Homo Sacer is a legal term that is also used in ethics. Deprivation of human rights and citizenship would allow the blood of Homo Sacer and other citizens could kill Homo Sacer without being responsible. In this situation, the Homo Sacer has fallen from the position of humanity and no law can defend his dignity and even his right to life. Homo Sacer can be considered an unholy sacrifice to preserve values. Basically, Agamben calls this situation where the Homo Sacer is suspended from all his rights until he is killed and punished, *“Exceptional Situation.”* Life in *“state of exception”* is a bare life that can be interrupted at any moment. In fact, this bare life is a defenseless life. By placing himself in the state of bare life, man becomes alienated from himself as a social being with basic rights and becomes a being who only deserves to die, which is also the case with Kolber too. Kolber is like a Homo Sacer who is insulted for his job, his job is mocked, he gets injured while going to Kolberi or falls on a mine and loses a part of his body, or even loses his life. This is the imminent future for Kolber.

A Kolber’s mother said: *“When my son Ibrahim was shot, we could not do anything for him. We did not have any legal or judicial support and we could not even complain to anyone.”*

No one is responsible for the conditions created for Kolbers and the official structure, which is itself responsible for the creation of such conditions, has withdrawn itself and even introduced them as smugglers and considers it permissible to deal with them violently. Kolber’s life is a bare life, which is deprived of any legal protection due to the abolition of rights, and no legal claim is realized in his case. His life path is such that there is no way forward and no way back. Inevitably, he steps on this path and submits to destiny. This fate is often predetermined, which is nothingness and destruction.

### 4.8 Structural dehumanization of Kolber’s position

All the behaviors that happen to the Kolbers in the border areas, both structurally and socially, dehumanize them. The result of these theoretical dehumanization is identifying a group of humans as *“imperfect humans”* and identifying another group as *“perfect humans.”* Based on his completeness, a perfect human being has legal privileges and finds legitimacy to exercise power over an imperfect human being, which is also an example of this practice in the case of the Kolbers, because from the point of view of the people and many officials, the Kolbers lacked a special social base in the society. And they are viewed as *“imperfect human beings”* or *“second and third class citizens.”*

A Kolber’s mother said: *“I told my son many times not to go to Kolberi. Carrying a load on a Kolber’s shoulder is the work of an animal, not a human being.”*

The representation of Kolberi in the dominant official and legal literature is as if their condition was the result of their own choice and they have other options, simply chose Kolberi and must be prepared for its consequences. This kind of visualization of Kolberi and Kolber allows any behavior and dealing with them and their death becomes not only an improvement of the situation, but an opportunity to attack them for taking up a threatening job. In many cases, the situation is created in such a way that the arrow of social criticism in the real and virtual environment is pointed towards a specific organization, while the situation is the result of a structural and fundamental decision or indecision in this case.

## 5 Discussion

Border regions are a product of colonial modernity, representing externally imposed divisions that disregard the cultural bonds of their inhabitants. Consequently, conflicts between states and cultures inevitably arise in these areas. The clash between cultural convergence across borderlines and state-sponsored violence is a defining feature of regions like Kurdistan. In this context, the ongoing dynamics in Kurdistan and other border regions can be understood as a distinct confrontation between state violence and the cultural realities shaping the sociology of the border residents.

The findings of this study shed light on the intricate dynamics and multifaceted challenges faced by Kolbers and their families in the border regions of western and northwestern Iran. The analysis uncovered several pervasive themes, each emblematic of the harsh realities endured by these individuals and their communities.

Firstly, the occurrence and exacerbation of physical and mental complications among Kolbers are evident. The hazardous nature of Kolberi work, undertaken in challenging terrains and often without proper safety measures, leads to severe physical injuries and mental distress. This aligns with theories on occupational hazards and risk exposure, reflecting the vulnerability of individuals engaged in perilous occupations (Wajnar and Swanson, 2007) that can not only affect their own lives but the lives of their surrounding people especially their family members.

Furthermore, the findings underscore the reproduction of poverty and misery as a driving force behind Kolberi. The lack of sustainable employment options, coupled with economic hardships and inadequate support systems, compels individuals to resort to Kolberi for their livelihood. This resonates with structural functionalism theories that emphasize the impact of socio-economic structures on shaping individual behaviors and choices (Durkheim, 1893).

The intensification of maternal sorrows and the hardships faced by Kolbers further accentuate the psychological toll on families. Mothers, in particular, experience profound anxiety and distress over the safety and well-being of their children engaged in Kolberi. This aligns with attachment theory, highlighting the emotional bonds and anxieties inherent in parent–child relationships, especially in contexts of high risk and adversity (Bowlby, 1969).

Moreover, the marginalization of education in border areas perpetuates the cycle of poverty. The lack of educational infrastructure and limited opportunities for quality education lead to high dropout rates among youngsters, further exacerbating socio-economic disparities. This aligns with critical theories on education, emphasizing the role of educational systems in either perpetuating or mitigating social inequalities (Apple, 1979).

The emergence of structural and environmental determinism in these border areas reinforces the notion of neglect and peripheralization by the central governing bodies. This echoes the concepts of structural violence and marginalization, elucidating how socio-political decisions can marginalize certain regions, leading to socio-economic disparities and vulnerability (Galtung, 1969).

The discussion also encompasses the dehumanization and existential precarity experienced by Kolbers. The portrayal of Kolbers as ‘bare life’ and their precarious existence without legal protections aligns with Agamben’s concepts, illustrating the stripping of basic human rights and the vulnerability of individuals within certain societal contexts (Agamben, 1998).

Overall, these findings underscore the complex interplay between socio-economic, political, and cultural factors in shaping the lives of Kolbers and their families. The theoretical underpinnings drawn from various sociological perspectives and critical theories elucidate the structural constraints and systemic injustices prevalent in these border regions. Addressing these issues necessitates a holistic approach centered on comprehensive development, socio-political empowerment, and a reconfiguration of policies to uplift marginalized communities from the grip of poverty and vulnerability.

In light of these circumstances, it is crucial to reevaluate the conventional notion of border regions. The current perspective, which considers borders as lifeless and seeks to discard their living nature, must be reconsidered. Contrary to this perspective, border regions emerge as vibrant life spaces intertwined with the cultures that grapple with seemingly inert borderlines, lacking social foundations. For the inhabitants, the border is not merely a dividing line; rather, it constitutes a life space integral to their way of life. Despite the prevailing objective and state-centric conception, the border is an intrinsic component of their existence on both sides.

Emphasizing the concept of doxa, Bourdieu proposes the assumed reality in which people are marginalized and experience the dominance of power as a result of the functioning of social structures. This domination crystallizes through symbolic violence and is applied to people. By experiencing this kind of violence, the border dwellers institutionalize the acceptance of the existing conditions and adapt themselves to it. This type of violence gradually manifests itself in the form of exclusion from the field of agency.

## 6 Conclusion

Kolberi, ingrained within the lives of those dwelling in the border regions of western and northwestern Iran, is not a chosen profession but a dire consequence of systemic destitution prevalent among the inhabitants, particularly the youth. The absence of essential infrastructure and sustainable employment opportunities in these areas shackles the livelihoods of those residing in the border villages to Kolberi. It is a stark reflection of the dearth of structural initiatives aimed at alleviating the pervasive poverty in these regions. For these border inhabitants, Kolberi is not a preference but rather the solitary avenue to subsistence and access to even the most basic facets of communal existence. Despite the numerous hazards and threats that loom over Kolbers, their occupation is born out of a necessity fueled by a landscape barren of employment prospects and devoid of fundamental structural support.

Amid this labyrinthine existence along the frontier, Kolbers’ mothers bear an overwhelming mental and psychological burden, envisioning an uncertain and harrowing future for their children. Their anguish surpasses all others, haunted by distressing images every time their children venture into the perilous unknown. Each report of a Kolber’s demise echoes the loss of their own offspring, and every grieving face mirrors their imminent fear and torment. These are the mothers of Kolbers, bracing themselves daily to face the stark possibility of embracing their children’s lifeless bodies in the relentless struggle for survival.

This profound emotional strain on Kolbers’ mothers, compounded by the arduous circumstances faced by border inhabitants, is a direct consequence of the region’s stunted development and prosperity. The lack of growth across economic, social, and infrastructural domains is the catalyst for the prevalence and exacerbation of these dire conditions. This absence of development has normalized death and pervasive personal and societal wounds in these areas, rendering them a stark and inevitable reality. The resurgence of life in these border regions necessitates a fundamental shift, one that embodies comprehensive development and instills a more compassionate perspective towards border residents, especially among governing authorities.

In light of these circumstances, the burden placed on existing organizations due to the situation not only fails to unravel the underlying causes of underdevelopment but further compounds the existing problems. Resolving this issue and alleviating the compulsion of border dwellers to resort to Kolberi hinges upon holistic development in these regions and the eradication of prejudiced perceptions toward border inhabitants from central authorities. It’s imperative that we envision a future where grieving mothers are not haunted by the tragic loss of their Kolber children.

Kolberi is a common job in the western and northwestern border areas of Iran, and it’s a big part of the daily life for the people there, especially the Kolbers and their families. While the Kolbers themselves might be able to handle the hardships that come with the job, mothers always carry the pain and stress of it after seeing what their loved ones go through. It’s not just the fear of something bad happening to their children, but the constant worry that it might happen at any moment. This waiting game is a never-ending source of stress for these mothers.

In addition to illuminating the pain and suffering experienced by Kolbers and their mothers, it is crucial to acknowledge the agency of the young individuals who, despite the considerable risks and dangers, actively strive to take control of their lives within the constraints of their circumstances. These young people, driven by a determination to secure their livelihoods, engage in Kolberi work as a form of agency, navigating the challenging terrains of the border region. The inclusion of this perspective adds a layer of complexity to the narrative, highlighting the resilience and resourcefulness of the youth in the face of adversity. By emphasizing their agency, the paper acknowledges the proactive steps taken by these individuals to shape their destinies, even when operating within highly circumscribed conditions. This dual focus on both the challenges faced and the agency exercised by the young Kolbers provides a more holistic understanding of their experiences and sheds light on their endeavors to assert control over their lives despite the formidable obstacles they encounter.

Overall, this study delves into the profound impact of Kolberi on the lives of individuals inhabiting the border regions of western and northwestern Iran. It sheds light on Kolberi as a consequence of systemic destitution, particularly among the youth, emphasizing its involuntary nature due to the lack of essential infrastructure and sustainable employment opportunities. The occupation becomes a desperate avenue for subsistence, reflecting the absence of structural initiatives aimed at mitigating pervasive poverty. The narrative extends to the emotional burden carried by Kolbers’ mothers, who face an uncertain future for their children amid the constant threat and peril. The dire conditions faced by border inhabitants, rooted in stunted development, necessitate a paradigm shift towards comprehensive development and a compassionate perspective from governing authorities. This study underscores the urgent need for holistic strategies to address the underlying causes of underdevelopment and dispel prejudiced perceptions, envisioning a future where grieving mothers are liberated from the haunting loss of their Kolber children.

The motherhood experiences of Kolbaran are a reflection of the underdevelopment of the region, which is the result of poverty, lack of educational infrastructure, lack of business opportunities, double marginality, and geographical determinism, which is based on the results of the studies of Salimi and Ghodrati (2020), Gallien and Weigand (2021), Daneshmehr and Mahmoudi (2023), Hernández (2023), and Sadeghi et al. (2014). What exists in addition to the mothers’ experience of these fears, is the difference in the views of the people and the government towards Kolberi phenomenon. People look at it as a job and means of livelihood, while the government considers it as a crime (trafficking) as what Lee (2015) concluded. This conceptual conflict has shown itself in the form of endless contradictions in the border field, which involves all the members of the Kolber families, including the mothers. May we aspire to witness a future where the painful narratives of Kolbers and their families are replaced by stories of development, opportunity, and humane compassion, nurturing a community where every life is valued and cherished.

## Data availability statement

The raw data supporting the findings of this study will be made accessible by the corresponding author upon request. Requests to access these datasets should be directed to HD, h.daneshmehr@uok.ac.ir.

## Ethics statement

The studies involving humans were approved by Faculty of Humanities and Social Sciences, University of Kurdistan, Sanandaj, Iran. The studies were conducted in accordance with the local legislation and institutional requirements. The participants provided their written informed consent to participate in this study. Written informed consent was obtained from the individual(s) for the publication of any potentially identifiable images or data included in this article.

## Author contributions

HD: Conceptualization, Data curation, Formal analysis, Investigation, Methodology, Project administration, Resources, Software, Supervision, Validation, Visualization, Writing – original draft, Writing – review & editing. KK: Formal analysis, Investigation, Methodology, Project administration, Writing – original draft, Writing – review & editing. PS: Data curation, Formal analysis, Investigation, Methodology, Resources, Visualization, Writing – review & editing. SR: Conceptualization, Formal analysis, Investigation, Methodology, Resources, Visualization, Writing – review & editing.

## Appendix

### Interview questions

1. How has your child’s Kolberi affected your daily life?
2. What does being Kolber’s mother mean to you?
3. What do you do to face the concerns that your child has when he goes to Kolberi?
4. How do you feel about your child’s Kolberi?
5. How does your time pass from the time your child goes to Kolberi until the time he returns home?
6. How do you feel when you hear or see about Kolberi from someone or on TV?
7. How do you feel when you hear the sad news of Kolberi’s death, his injury, or his fall from the mountain?
8. If you liked to tell me a story about the day your child went to Kolberi and you were unaware of it for a few days or hours, what would be your worries? And what would you do?
9. How did you feel about Kolberi before your child became Kolber, and how do you feel now?
10. What did you think about your child’s future? What did you imagine? What did you wish for your child’s future?
11. What does Kolberi evoke for you? What is it comparable to?
12. How has Kolberi affected the education of your children?
13. What is the difference between the children of Kolbers and those who do not do Kolberi?
14. Does the social suffering and high stress of mothers harm their children?
15. How does your child’s Kolberi affect the society?
